# Supplementary material for: Single nucleotide polymorphism determines constitutive versus inducible type VI secretion in Vibrio cholerae
Source: ISME J. 2022 Apr 11;16(7):1868–72. doi: 10.1038/s41396-022-01234-7 (PMC9213491; doi:10.1038/s41396-022-01234-7)
Supplement: Supplementary file 1 — Supplementary material [file 41396_2022_1234_MOESM1_ESM.pdf]

SUPPLEMENTARY INFORMATION

Single nucleotide polymorphism determines constitutive versus inducible type VI secretion in *Vibrio cholerae*

Natália C. Drebes Dörr, Alexis Proutière, Milena Jaskólska, Sandrine Stutzmann, Loriane Bader, and Melanie Blokesch

Content – Supplementary Information

- Supplementary Material and Methods
- Supplementary Figures S1 and S2
- Supplementary Tables S1, S2, S3, S4
- Supplementary References

Supplementary Material and Methods

Bacterial strains and growth conditions

Bacterial strains used in this study are listed in Supplementary Table S3. Unless otherwise stated, strains were grown aerobically in Lysogeny broth (LB; 10 g/L of tryptone, 5 g/L of yeast extract, 10 g/L of sodium chloride; Carl Roth) or on LB agar plates at 30°C or 37°C. Half-concentrated defined artificial seawater medium (0.5×DASW) containing HEPES (4-(2-hydroxyethyl)-1-piperazineethanesulfonic acid; Sigma-Aldrich) and vitamins [1] or 0.5× HW Marine Mix (Wiegandt, Germany) were used for growth on chitin (see below for natural transformation-based strain construction). Thiosulfate citrate bile salts sucrose (TCBS, Sigma-Aldrich) agar was used to counter-select *Escherichia coli* following bacterial mating. Counter-selection based on *sacB* was performed on NaCl-free medium containing 10% sucrose. When required, the following antibiotics were added at their given concentrations: kanamycin (75 μg/mL), chloramphenicol (2.5 μg/mL), streptomycin (100 μg/mL), gentamicin (50 μg/mL), and ampicillin (100 μg/mL). To induce expression from the P_{BAD} promoter (inside TnTfoX, TnQstR, and TnTfoY), culture media were supplemented with 0.2% L-arabinose.

Recombinant DNA techniques and genetic engineering

DNA manipulations/cloning was carried out using standard methods. PCR amplifications were performed using GoTaq (Promega), Pwo (Roche), or Expand High Fidelity (Roche) polymerases according to the suppliers' recommendations. Genetically modified loci were checked by colony PCR and Sanger sequencing (Microsynth, Switzerland).

V. cholerae were genetically engineered using a set of different methods. Natural transformation on chitin flakes and FLP-recombination (TransFLP; [2, 3]) were used to integrate the *vipA-sfGFP* translational fusion construct allele into the chromosome of diverse strains as reported [4]. Natural transformation on chitin flakes using PCR fragments was also

used for the hybrid strain library construction (see details below). All remaining mutations and deletions were done by allelic exchange using the counter-selectable plasmids pGP704-Sac28 and pGP704-Sac-Kan [5, 6].

Construction of a pandemic/non-pandemic hybrid strain library

The kanamycin-resistance marker *aph* was integrated by natural transformation into the genome of pandemic *V. cholerae* strain A1552. To do so, amplified PCR fragments served as transforming material, whereby each fragment combined an upstream region, *aph* preceded by its promoter, and a downstream region (fused using overlapping PCR). The PCR fragments were prepared to generate 40 transformants (*aph*#1 to *aph*#40) whereby each strain contains the *aph* cassette at a different location (roughly every 100 kb). Genomic DNA (gDNA) of these 40 donor strains was isolated from 2 ml of an overnight culture using 100/G Genomic-tips together with a Genomic DNA buffer set (Qiagen) as described in the manufacturer's instructions.

To generate the pandemic/non-pandemic hybrid strain library, the acceptor strain ATCC25872 was grown in 40 parallel tubes on chitin flakes and each donor strain-derived gDNA was added to one of those tubes. After incubation at 30°C for 30 h, bacteria were selected on LB plates containing kanamycin and 20 transformants were isolated and stocked from each independent reaction, resulting in 800 strains in total.

After the first screen of the 800 strains library, the reverse experiment from what was described above was performed, generating a set of ATCC25872 strains carrying *aph* at 40 different genomic locations. However, transformation of A1552 as acceptor strain was only performed using gDNA of strain ATCC25872-*aph*#32 as transforming material. 20 transformants of this reaction were isolated and stocked and further explored for their T6SS activity. Based on the screening result and the proximity of *aph*#32 to the large T6SS cluster,

an additional construct was generated in both strains (A1552-*aph*#42 and ATCC25872-*aph*#42) and their gDNA used to transform in each case the opposite strain to obtain 2 x 20 additional hybrid clones.

Phenotypic screening of the hybrid strain library

The initial library was screened for the strains' T6SS activities using a fluorescence-based *E. coli* killing experiment, which was adapted from [7]. Briefly, 200 µL of the predator hybrid strain were mixed with 40 µL of GFP-labelled *E. coli* prey (strain MC4100-TnGFP) in 96-well plates and 5 µL of the mixtures were spotted onto LB plates. The donor (A1552; T6SS OFF) and acceptor (ATCC25872; T6SS ON) strains served as controls. After 4 h incubation at 37°C, the plates were observed under a stereo microscope (Leica EL6000) equipped with a green fluorescence (FITC) filter cube. Prey survival was scored based on their maintained GFP signal. To properly quantify T6SS activity, all *aph*#32 and *aph*#42 transformants were rechecked using the standard interbacterial killing assay described below with *E. coli* TOP10-TnGFP (Cm^R) as prey.

Interbacterial killing assay

Bacterial killing was assessed following a previously established assay [4]. Briefly, the respective predator and the *E. coli* prey were mixed at a ratio of 10:1 and spotted onto filters on prewarmed LB agar plates (containing 0.2% arabinose where indicated in the figure legend). After 4 h of incubation at 37°C, the bacteria were resuspended, serially diluted, and spotted onto antibiotic-containing (streptomycin, kanamycin, or chloramphenicol) LB agar plates to enumerate colony-forming units (shown as log-transformed CFU/mL in the graphs). Experiments were performed at least three independent times. Statistically significance was determined using using GraphPad Prism 9.1.1 (for macOS) on log-transformed data [8] using

a one- or two-way ANOVA followed by a Šídák's multiple comparisons test, as indicated in the figure legends. If no prey bacteria were recovered, the detection limit was used to calculate the mean of the independent experiments and to perform the statistical analysis.

Imaging of T6SS sheath structure

To image T6SS sheath structures in strains carrying the translational fusion *vipA-sfGFP*, the bacteria were mounted on microscope slides coated with a thin agarose pad (1.2% in 0.5X PBS), covered with a coverslip, and observed in the phase contrast and epifluorescence mode (green channel) using a Zeiss LSM 700 inverted confocal laser scanning microscope with an attached HXP 120 light unit (Zeiss, Feldbach, Switzerland). Images were adjusted for contrast and brightness and cropped using the Fiji software [9]. The images are overlays of the Ph and GFP channels and representative of at least three biologically independent replicates.

Whole-genome sequencing and *de novo* assembly of strain ATCC25872

Bacterial growth and gDNA extraction were done as described [10]. DNA sample preparation and genome sequencing was performed by the Genomic Technology Facility of the University of Lausanne (Switzerland). Briefly, the DNA sample was sheared in Covaris g-TUBEs resulting in fragments with a mean length of 20 kb. The DNA library was prepared using the PacBio SMRTbell template prep kit 1 (Pacific Biosciences) according to the manufacturer's recommendations and size selected on a BluePippin system (Sage Science, Inc.) for molecules larger than 15 kb. The library was sequenced on a PacBio system within one single-molecule real-time (SMRT) cell with P6/C4 chemistry and MagBeads at a movie length of 360 min. The genome was *de novo* assembled using the protocol RS_HGAP_Assembly.3 in SMRT Pipe 2.3.0 and circularized using the Minimus assembler of the AMOS software package 3.1.0 using default parameters [11]. The assembled genome was annotated using Prokka 1.12 [12]

but due to incompatibly with the NCBI database, reannotated using their Prokaryotic Genome Annotation Pipeline (PGAP) during data submission. The data are available under BioSample SAMN13736322 and BioProject PRJNA599000 and the NCBI accession numbers CP047305 (chromosome 1) and CP047306 (chromosome 2). Details on the genome sequencing and assembly are provided in Supplementary Table S4.

Expression profiling by RNA sequencing

Overnight cultures of strains A1552 and ATCC25872 and their SNP45-converted derivatives were back-diluted 1:100 in LB medium and grown for 3 h at 30°C with agitation. Cells were harvested by centrifugation at 4°C and washed with PBS buffer, followed by lysis with Tri Reagent (Sigma-Aldrich) and shock freezing in a dry-ice ethanol bath. The samples were stored at -80 °C prior to processing. RNA preparation and DNase treatment were performed as previously described [13]. After DNase treatment, an additional purification step was performed using the GenElute Mammalian Total RNA miniprep kit (Sigma-Aldrich).

Downstream processing of the samples was performed by Microsynth AG (Balgach, Switzerland) who also analyzed the data. Briefly, Illumina's TruSeq Stranded Total RNA Library Prep Gold kit including ribodepletion was used to construct libraries from total RNA. Subsequently, an Illumina NextSeq 550 platform and a high output v2.5 kit were used to sequence the library. Single end reads (1x75bp), which passed Illumina's chastity filter, were demultiplexed and trimmed off the Illumina adaptors using Illumina's bcl2fastq software version v2.20.0.422 (no further refinement or selection). The quality of the reads in fastq format was checked with the FastQC software (version 0.11.8) (http://www.bioinformatics.babraham.ac.uk/projects/fastqc/). The reads were mapped to the reference genomes (A1552 chromosome 1 [CP028894] and 2 [CP028895]; ATCC25872 chromosome 1 [CP047305] and 2 [CP047306]) via bowtie2 (version 2.3.5.1) [14] in local

mapping mode with very sensitive pre-settings. To count the uniquely mapped reads to annotated genes, the software htseq-count (HTSeq version 0.11.2) [15] was used. Normalization of the raw counts and differential gene expression analysis was carried out with help of the R software package DESeq2 (version 1.22.2) [16]. Data were visualized using the Integrative Genomics Viewer [17].

Quantitative Reverse Transcription PCR (qRT-PCR)

To analyze gene expression using quantitative reverse transcription PCR (qRT-PCR), overnight cultures were back-diluted 1:100 in LB medium and grown for 3 h at 30°C with agitation. RNA purification, DNase treatment, cDNA synthesis and qPCR followed a previously established protocol [13]. Samples were analyzed on a LightCycler Real-Time PCR System (LightCycler Nano or LightCycler 96; Roche) using the standard curve method. Expression values are presented relative to the transcript levels of the reference gene *gyrA*. Each experiment was performed two independent times.

Western blotting

To check the production of the inner tube T6SS protein Hcp, cell lysates were prepared as described previously [18]. In brief, overnight cultures were back-diluted 1:100 in LB medium and grown with agitation at 30°C for 3 h. Cells were harvested by centrifugation and the bacterial pellet was resuspended in 2× Laemmli buffer (Sigma-Aldrich), adjusting for the total number of bacteria according to the cultures' optical density at 600nm (OD_{600}) values. To check for T6SS-secreted Hcp, 1.5 ml of the culture supernatant was filter sterilized (0.2 µm filter; VWR) and the proteins were precipitated using trichloroacetic acid (TCA). The precipitated proteins were washed with acetone before resuspension in 30 µl of 2× Laemmli buffer (Sigma-Aldrich). All samples were heated at 95°C for 15 min.

Proteins were separated by sodium dodecyl sulfate (SDS) polyacrylamide gel electrophoresis (PAGE) using 15% gels and then blotted as previously described [13]. Primary antibodies against Hcp (raised against synthetic peptides, Eurogentec #1510528; [18]) were diluted 1:5,000 while the Anti-rabbit IgG HRP (Sigma-Aldrich, A9169) secondary antibody was diluted 1:20,000. Loading controls were performed using the anti-Sigma70-horseradish peroxidase (HRP) conjugate (BioLegend, 663205) at a 1:10,000 dilution. Lumi-Light^{PLUS} Western Blotting Substrate (Roche, Switzerland) served as the HRP substrate. The signal was detected using a ChemiDoc XRS+ station (BioRad).

Data availability

The RNAseq dataset (for strains A1552, SNP45-converted A1552, ATCC25872, and SNP45-converted ATCC25872) is accessible through the GEO Series accession number GSE196165.

Supplementary Figures

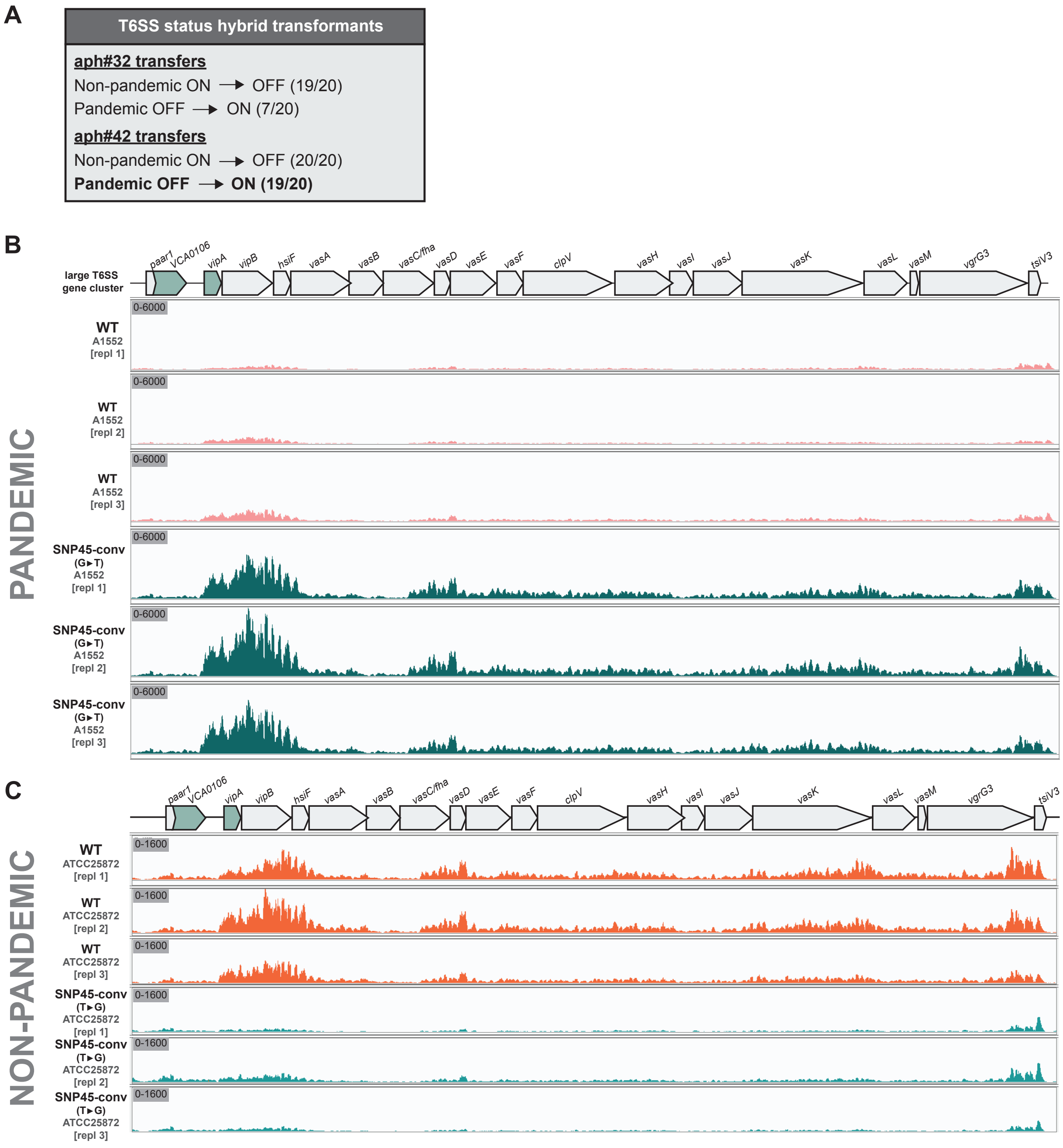

Figure S1. SNP45-conversion changes T6SS-related phenotypes. (A) Hybrid strains with changed T6SS activity status. Table summarizes the number of *aph*#32 and *aph*#42 transformants that changed their T6SS phenotype compared to the parental strain (non-pandemic strain ATCC25872 or pandemic strain A1552). The single non-converted transformed clone #17 in Fig. 1B was part of the set highlighted in bold. **(B-C)** SNP-conversion leads to changed transcript abundance. RNA-sequencing data with normalized reads matched to the large T6SS cluster region (*paar1* to *tsiV3*) of *V. cholerae* A1552 (B) or ATCC25872 (C). Three biologically independent replicates of WT and their SNP45-converted derivatives are shown. The scales of the *Y*-axis are shown on the upper left of each row. See Supplementary Tables S1 and S2 for details.

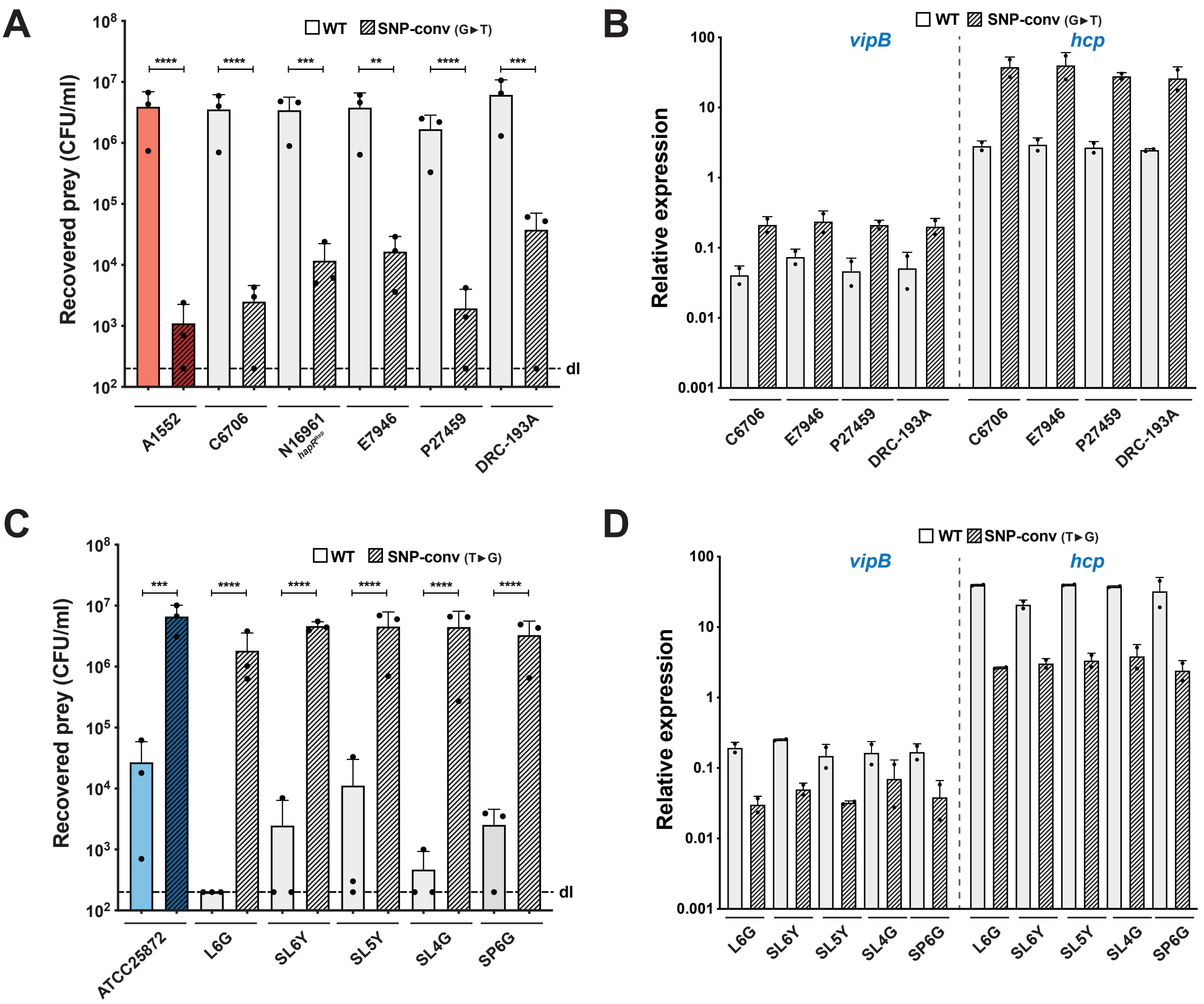

Figure S2. SNP45-conversion changes T6SS status in pandemic and environmental strains. SNP45-conversion inverts the ability of strains to kill prey bacteria, as assessed in an *E. coli* killing assay. Results for WT and SNP45-converted derivatives of six well-studied pandemic strains (N16961 derivative is QS-repaired; [19]) **(A)** or five environmental isolates (with ATCC25872 as control) **(C)** are shown. d.l., detection limit. Bar plots represent the average of three independent biological replicates as shown by the individual dots (±SD). Statistically analysis was done using one-way ANOVA followed by a Šídák's multiple comparisons test whereby WT and SNP-converted derivatives were compared. **, $p < 0.01$; ***, $p < 0.001$; ****, $p < 0.0001$. **(B, D)** A selection of the strains from (A, C) were scored for the expression of two conserved T6SS genes (*vipB* and *hcp*) using qRT-PCR. Data represent the average of two independent biological replicates (±SD). Details are provided in the Supplementary Methods section.

Table S1. RNA-seq expression data for the T6SS gene cluster in WT and SNP45-converted (G->T) strain A1552. Normalized read counts are listed.

cluster names / comments	gene names	ID (locus tag) in strain A1552*	Homologous locus tag in strain N16961#	A1552 (Exp1)	A1552 (Exp2)	A1552 (Exp3)	AVG - A1552	A1552-SNP45 converted (Exp1)	A1552-SNP45 converted (Exp2)	A1552-SNP45 converted (Exp3)	AVG - A1552-SNP45 converted	Fold induction [A1552-SNP45 converted / A1552]
major T6SS regulators	*tfoX*	A1552VC_00900	VC1153	7070.354	4314.475	5845.282	5743.37	4115.60	4237.94	5746.86	4700.14	**0.82**
	qstR	A1552VC_00153	VC0396	149.906	232.308	193.050	191.75	219.06	233.16	236.88	229.70	**1.20**
	tfoY	A1552VC_01512	VC1722	8158.21	5470.85	5678.76	6435.94	7128.59	5720.72	5611.63	6153.65	**0.96**
Auxiliary cluster 1	*hcp1*	A1552VC_01148	VC1415	3912.69	3666.59	4261.83	3947.04	70511.89	79856.98	81253.87	77207.58	**19.56**
	vgrG1	A1552VC_01149	VC1416	1043.84	1071.20	1272.51	1129.18	7298.36	14019.01	9719.39	10345.59	**9.16**
	tap1	A1552VC_01150	VC1417	812.80	745.97	815.67	791.48	4627.65	7497.69	5053.46	5726.27	**7.23**
	tseL	A1552VC_01151	VC1418	3398.34	2551.52	2616.49	2855.45	14387.70	21816.35	14907.18	17037.08	**5.97**
	tsiV1	A1552VC_01152	VC1419	722.03	503.33	553.36	592.91	2757.42	4232.48	3178.38	3389.43	**5.72**
Auxiliary cluster 2	*hcp2*	A1552VC_A02810	VCA0017	4229.01	4177.67	5050.24	4485.64	77191.41	90673.62	95681.83	87848.96	**19.58**
	vgrG2	A1552VC_A02811	VCA0018	226.92	331.68	350.73	303.11	2760.16	4041.21	3289.11	3363.49	**11.10**
	vasW	A1552VC_A02812	VCA0019	301.19	313.62	333.78	316.20	2775.68	3845.39	2965.10	3195.39	**10.11**
	vasX	A1552VC_A02813	VCA0020	2492.02	2071.41	2094.81	2219.42	17124.13	22817.32	17103.55	19015.00	**8.57**
	tsiV2	A1552VC_A02814	VCA0021	1009.46	633.68	723.57	788.90	4994.58	4717.02	4508.00	4739.87	**6.01**
Large/major cluster	*paar1*	A1552VC_A02890	VCA0105	375.45	304.58	338.21	339.41	648.05	574.71	642.57	621.78	**1.83**
	VCA0106	A1552VC_A02891	VCA0106	515.73	610.45	674.94	600.37	1560.81	1640.35	2026.65	1742.60	**2.90**
	vipA	A1552VC_A02892	VCA0107	899.44	2086.90	2008.60	1664.98	7895.30	10908.63	10440.01	9747.98	**5.85**
	vipB	A1552VC_A02893	VCA0108	4556.33	8768.34	8324.72	7216.46	37664.70	51986.55	43125.08	44258.77	**6.13**
	hsiF	A1552VC_A02894	VCA0109	1104.36	1360.29	1393.35	1286.00	7502.82	8755.51	7003.88	7754.07	**6.03**
	vasA	A1552VC_A02895	VCA0110	1272.14	1662.29	1589.34	1507.93	8117.10	10363.06	7957.75	8812.64	**5.84**
	vasB	A1552VC_A02896	VCA0111	374.08	383.31	478.20	411.86	2024.48	2205.04	2086.55	2105.36	**5.11**
	fha	A1552VC_A02897	VCA0112	1466.06	1377.07	1244.51	1362.54	7132.24	8243.64	5585.31	6987.06	**5.13**
	vasD	A1552VC_A02898	VCA0113	913.19	809.21	780.30	834.23	4111.03	5138.72	3662.13	4303.96	**5.16**
	vasE	A1552VC_A02899	VCA0114	1548.57	1433.86	1483.24	1488.56	7876.13	8273.69	6194.31	7448.04	**5.00**
	vasF	A1552VC_A02900	VCA0115	942.07	724.03	820.09	828.73	4455.14	4581.32	3596.78	4211.08	**5.08**
	clpV	A1552VC_A02901	VCA0116	2731.32	2480.53	2695.33	2635.73	12643.44	13948.88	11507.34	12699.89	**4.82**
	vasH	A1552VC_A02902	VCA0117	1860.76	1533.23	1728.61	1707.53	7732.83	8198.10	7034.73	7655.22	**4.48**
	vasI	A1552VC_A02903	VCA0118	264.06	234.89	269.68	256.21	1022.28	1092.05	953.88	1022.74	**3.99**
	vasJ	A1552VC_A02904	VCA0119	1009.46	961.50	1147.98	1039.65	4006.07	5063.13	4443.56	4504.25	**4.33**
	vasK	A1552VC_A02905	VCA0120	5391.13	5006.24	5057.61	5151.66	19344.86	26136.27	21373.76	22284.96	**4.33**
	vasL	A1552VC_A02906	VCA0121	2178.46	1299.63	1288.72	1588.94	6452.24	6615.13	5387.46	6151.61	**3.87**
	vasM	not annotated	VCA0122	-	-	-	-	-	-	-	-	**-**
	vgrG3	A1552VC_A02907	VCA0123	4535.70	3550.44	3404.90	3830.35	11925.10	16847.95	12063.69	13612.25	**3.55**
	tsiV3	A1552VC_A02908	VCA0124	1985.92	1045.39	1053.67	1361.66	3814.39	3902.77	3242.82	3653.33	**2.68**
Auxiliary cluster 3	*paar2*	A1552VC_A03052	VCA0284	980.58	447.84	573.25	667.22	1506.95	1676.78	1873.27	1685.67	**2.53**
	tseH	A1552VC_A03053	VCA0285	629.88	308.45	369.15	435.83	1376.43	2073.89	2288.04	1912.78	**4.39**
	tsiH	A1552VC_A03054	VCA0286	413.96	243.92	313.15	323.68	784.97	986.39	1200.74	990.70	**3.06**

* according to Matthey, Drebes Dörr *et al.* , 2018 [21] - genome accession numbers CP028894 and CP028895.
# according to Heidelberg *et al.* , 2000 [30] - genome accession numbers NC_002505 and NC_002506

Table S2. RNA-seq expression data for the T6SS gene cluster in WT and SNP45-converted (T->G) strain ATCC25872. Normalized read counts are listed.

cluster names / comments	gene names	ID (locus tag) in strain ATCC25872*	Newly assigned locus tag in strain ATCC25872$	Homologous locus tag in strain N16961#	ATCC25872 (Exp1)	ATCC25872 (Exp2)	ATCC25872 (Exp3)	AVG - ATCC25872	ATCC25872-SNP45 converted (Exp1)	ATCC25872-SNP45 converted (Exp2)	ATCC25872-SNP45 converted (Exp3)	AVG - ATCC25872-SNP45 converted	Fold induction [ATCC25872-SNP45 converted / ATCC25872]
major T6SS regulators	***tfoX***	AT72VC_01697	GTH07_08410	VC1153	5746.36	4043.40	5322.23	5037.33	4000.35	3001.05	4385.87	3795.76	**0.75**
	qstR	AT72VC_02419	GTH07_11940	VC0396	165.22	245.69	257.30	222.74	256.06	216.54	243.34	238.65	**1.07**
	tfoY	AT72VC_01140	GTH07_05665	VC1722	5202.51	5065.44	5242.38	5170.11	6040.69	5384.32	4998.28	5474.43	**1.06**
Auxiliary cluster 1	***hcp1***	AT72VC_01454	GTH07_07215	VC1415	19554.14	19652.54	18391.42	19199.37	684.38	509.52	569.34	587.74	**0.03**
	vgrG1	AT72VC_01453	GTH07_07210	VC1416	5045.15	5650.89	5372.93	5356.33	486.51	571.30	484.34	514.05	**0.10**
	tap1	AT72VC_01452	GTH07_07205	VC1417	3486.37	3724.64	3039.46	3416.82	423.66	503.78	385.38	437.61	**0.13**
	tseL	AT72VC_01451	GTH07_07200	VC1418	11535.01	10718.16	8489.71	10247.63	1822.68	1887.12	1532.20	1747.33	**0.17**
	tsiV1	AT72VC_01450	GTH07_07195	VC1419	2237.37	2221.27	1737.74	2065.46	434.14	364.94	303.88	367.65	**0.18**
Auxiliary cluster 2	***hcp2***	AT72VC_A00658	GTH07_13450	VCA0017	34594.21	31399.13	28601.12	31531.49	1070.80	894.84	908.14	957.93	**0.03**
	vgrG2	AT72VC_A00659	GTH07_13455	VCA0018	1691.55	1789.25	2041.94	1840.92	197.86	164.32	140.88	167.69	**0.09**
	vasW	AT72VC_A00660	GTH07_13460	VCA0019	2132.14	2152.76	1951.95	2078.95	268.86	205.08	174.64	216.20	**0.10**
	vasX	AT72VC_A00661	GTH07_13465	VCA0020	13234.43	12175.86	9992.97	11801.09	1497.95	1267.42	1102.58	1289.32	**0.11**
	tsiV2	AT72VC_A00662	GTH07_13470	VCA0021	3450.96	2861.52	2849.34	3053.94	600.58	518.43	468.04	529.02	**0.17**
Large/major cluster	***paar1***	AT72VC_A00737	GTH07_13830	VCA0105	384.53	358.95	385.32	376.27	290.98	307.62	292.24	296.94	**0.79**
	VCA0106	AT72VC_A00738	GTH07_13835 (diff. start codon)	VCA0106	706.12	782.74	846.69	778.52	484.19	574.48	548.38	535.68	**0.69**
	vipA	AT72VC_A00739	GTH07_13840	VCA0107	1544.03	3053.33	2929.19	2508.85	442.29	544.55	525.09	503.97	**0.20**
	vipB	AT72VC_A00740	GTH07_13845	VCA0108	8279.75	12722.05	10783.89	10595.23	1395.53	2029.79	1857.04	1760.78	**0.17**
	hsiF	AT72VC_A00741	GTH07_13850	VCA0109	2259.01	2625.88	2116.72	2333.87	246.75	345.20	331.82	307.92	**0.13**
	vasA	AT72VC_A00742	GTH07_13855	VCA0110	2802.86	3578.50	2946.94	3109.43	366.63	570.66	497.15	478.15	**0.15**
	vasB	AT72VC_A00743	GTH07_13860	VCA0111	733.66	652.13	803.59	729.80	97.77	135.66	140.88	124.77	**0.17**
	fha	AT72VC_A00744	GTH07_13865	VCA0112	2701.57	2296.16	1802.38	2266.70	342.19	445.83	376.06	388.03	**0.17**
	vasD	AT72VC_A00745	GTH07_13870	VCA0113	1634.51	1662.30	1131.88	1476.23	251.40	259.22	222.38	244.33	**0.17**
	vasE	AT72VC_A00746	GTH07_13875	VCA0114	2605.19	2778.41	2040.67	2474.76	425.99	527.35	437.77	463.70	**0.19**
	vasF	AT72VC_A00747	GTH07_13880	VCA0115	1576.49	1380.07	1097.65	1351.40	235.11	280.23	223.54	246.30	**0.18**
	clpV	AT72VC_A00748	GTH07_13885	VCA0116	4491.46	4472.67	3551.53	4171.89	890.39	1061.07	867.39	939.62	**0.23**
	vasH	AT72VC_A00749	GTH07_13890	VCA0117	3205.10	3151.97	2592.04	2983.03	665.76	737.53	649.67	684.32	**0.23**
	vasI	AT72VC_A00750	GTH07_13895	VCA0118	432.72	396.39	394.19	407.77	83.80	120.37	121.09	108.42	**0.27**
	vasJ	AT72VC_A00751	GTH07_13900	VCA0119	1448.64	1455.88	1339.75	1414.75	301.45	376.41	341.14	339.66	**0.24**
	vasK	AT72VC_A00752	GTH07_13905	VCA0120	10261.43	10150.05	8146.22	9519.23	1559.64	2015.78	1793.00	1789.47	**0.19**
	vasL	AT72VC_A00753	GTH07_13910	VCA0121	3757.80	2878.88	2001.38	2879.35	523.76	552.83	472.70	516.43	**0.18**
	vasM	not annotated	not annotated	VCA0122	-	-	-	-	-	-	-	-	**-**
	vgrG3	AT72VC_A00754	GTH07_13915	VCA0123	7884.40	6577.94	5367.86	6610.07	1783.11	1917.06	1458.85	1719.67	**0.26**
	tsiV3	AT72VC_A00755	GTH07_13920	VCA0124	2256.06	1586.49	1221.87	1688.14	669.25	664.28	561.19	631.57	**0.37**
Auxiliary cluster 3	***paar2***	AT72VC_A00904	GTH07_14650	VCA0284	973.63	926.14	866.97	922.24	684.38	644.54	470.37	599.76	**0.65**
	tseH	AT72VC_A00905	GTH07_14655	VCA0285	765.13	906.96	788.38	820.16	543.55	445.83	284.09	424.49	**0.52**
	tsiH	AT72VC_A00906	GTH07_14660 (diff. start codon)	VCA0286	472.06	569.02	487.99	509.69	385.25	277.05	224.71	295.67	**0.58**

* according to Prokka annotation used for RNA-seq read mapping in this work.

$ according to NCBI's PGAP annotation - genome accession numbers CP047305 and CP047306.

according to Heidelberg *et al.*, 2000 [30] - genome accession numbers NC_002505 and NC_002506.

Table S3. *Vibrio cholerae* and *Escherichia coli* strains and plasmids used in this study.

Strain names	Genotype / description*	Internal strain number	Reference
V. cholerae			
A1552	Wild-type, O1 El Tor Inaba, isolated in 1991 in Peru; Rif^R.	MB_1	[20, 21]
ATCC25872	Wild-type, non-O1 strain (O37); isolated in 1965 in Czechoslovakia; intermediate $Strep^R$	MB_276	[22]
A1552-*aph*#1	A1552 with *aph* cassette in position #1 on chr 1; Rif^R, Kan^R	MB_6911	This study
A1552-*aph*#2	A1552 with *aph* cassette in position #2 on chr 1; Rif^R, Kan^R	MB_6912	This study
A1552-*aph*#3	A1552 with *aph* cassette in position #3 on chr 1; Rif^R, Kan^R	MB_6913	This study
A1552-*aph*#4	A1552 with *aph* cassette in position #4 on chr 1; Rif^R, Kan^R	MB_6914	This study
A1552-*aph*#5	A1552 with *aph* cassette in position #5 on chr 1; Rif^R, Kan^R	MB_6915	This study
A1552-*aph*#6	A1552 with *aph* cassette in position #6 on chr 1; Rif^R, Kan^R	MB_6916	This study
A1552-*aph*#7	A1552 with *aph* cassette in position #7 on chr 1; Rif^R, Kan^R	MB_6917	This study
A1552-*aph*#8	A1552 with *aph* cassette in position #8 on chr 1; Rif^R, Kan^R	MB_6918	This study
A1552-*aph*#9	A1552 with *aph* cassette in position #9 on chr 1; Rif^R, Kan^R	MB_6919	This study
A1552-*aph*#10	A1552 with *aph* cassette in position #10 on chr 1; Rif^R, Kan^R	MB_6920	This study
A1552-*aph*#11	A1552 with *aph* cassette in position #11 on chr 1; Rif^R, Kan^R	MB_6921	This study
A1552-*aph*#12	A1552 with *aph* cassette in position #12 on chr 1; Rif^R, Kan^R	MB_6922	This study
A1552-*aph*#13	A1552 with *aph* cassette in position #13 on chr 1; Rif^R, Kan^R	MB_6923	This study
A1552-*aph*#14	A1552 with *aph* cassette in position #14 on chr 1; Rif^R, Kan^R	MB_6924	This study
A1552-*aph*#15	A1552 with *aph* cassette in position #15 on chr 1; Rif^R, Kan^R	MB_6925	This study
A1552-*aph*#16	A1552 with *aph* cassette in position #16 on chr 1; Rif^R, Kan^R	MB_6926	This study
A1552-*aph*#17	A1552 with *aph* cassette in position #17 on chr 1; Rif^R, Kan^R	MB_6927	This study
A1552-*aph*#18	A1552 with *aph* cassette in position #18 on chr 1; Rif^R, Kan^R	MB_6928	This study
A1552-*aph*#19	A1552 with *aph* cassette in position #19 on chr 1; Rif^R, Kan^R	MB_6929	This study
A1552-*aph*#20	A1552 with *aph* cassette in position #20 on chr 1; Rif^R, Kan^R	MB_6930	This study
A1552-*aph*#21	A1552 with *aph* cassette in position #21 on chr 1; Rif^R, Kan^R	MB_6931	This study
A1552-*aph*#22	A1552 with *aph* cassette in position #22 on chr 1; Rif^R, Kan^R	MB_6932	This study
A1552-*aph*#23	A1552 with *aph* cassette in position #23 on chr 1; Rif^R, Kan^R	MB_6933	This study
A1552-*aph*#24	A1552 with *aph* cassette in position #24 on chr 1; Rif^R, Kan^R	MB_6934	This study
A1552-*aph*#25	A1552 with *aph* cassette in position #25 on chr 1; Rif^R, Kan^R	MB_6935	This study
A1552-*aph*#26	A1552 with *aph* cassette in position #26 on chr 1; Rif^R, Kan^R	MB_6936	This study
A1552-*aph*#27	A1552 with *aph* cassette in position #27 on chr 1; Rif^R, Kan^R	MB_6937	This study
A1552-*aph*#28	A1552 with *aph* cassette in position #28 on chr 1; Rif^R, Kan^R	MB_6938	This study
A1552-*aph*#29	A1552 with *aph* cassette in position #29 on chr 1; Rif^R, Kan^R	MB_6939	This study
A1552-*aph*#30	A1552 with *aph* cassette in position #30 on chr 1; Rif^R, Kan^R	MB_6940	This study
A1552-*aph*#31	A1552 with *aph* cassette in position #31 on chr 2; Rif^R, Kan^R	MB_6941	This study
A1552-*aph*#32	A1552 with *aph* cassette in position #32 (15kb upstream of T6SS large cluster) on chr 2; Rif^R, Kan^R	MB_6942	This study
A1552-*aph*#33	A1552 with *aph* cassette in position #33 on chr 2; Rif^R, Kan^R	MB_6943	This study
A1552-*aph*#34	A1552 with *aph* cassette in position #34 on chr 2; Rif^R, Kan^R	MB_6944	This study
A1552-*aph*#35	A1552 with *aph* cassette in position #35 on chr 2; Rif^R, Kan^R	MB_6945	This study
A1552-*aph*#36	A1552 with *aph* cassette in position #36 on chr 2; Rif^R, Kan^R	MB_6946	This study
A1552-*aph*#37	A1552 with *aph* cassette in position #37 on chr 2; Rif^R, Kan^R	MB_6947	This study
A1552-*aph*#38	A1552 with *aph* cassette in position #38 on chr 2; Rif^R, Kan^R	MB_6948	This study
A1552-*aph*#39	A1552 with *aph* cassette in position #39 on chr 2; Rif^R, Kan^R	MB_6949	This study

A1552-*aph*#40	A1552 with *aph* cassette in position #40 on chr 2; Rif^R, Kan^R	MB_6950	This study
A1552-*aph*#42	A1552 with *aph* cassette in position #42 on chr 2 (upstream of *paar1/VCA0105*); Rif^R, Kan^R	MB_7922	This study
ATCC25872-*aph*#1^{A1552} hybrid clones 1-20	ATCC25872 transformed with gDNA of strain A1552-*aph*#1 (#6911). 20 hybrid transformants selected; intermediate $Strep^R$, Kan^R	MB_7001 to 7020	This study
ATCC25872-*aph*#2^{A1552} hybrid clones 1-20	ATCC25872 transformed with gDNA of strain A1552-*aph*#2 (#6912). 20 hybrid clones selected; intermediate $Strep^R$, Kan^R	MB_7021 to 7040	This study
ATCC25872-*aph*#3^{A1552} hybrid clones 1-20	ATCC25872 transformed with gDNA of strain A1552-*aph*#3 (#6913). 20 hybrid clones selected; intermediate $Strep^R$, Kan^R	MB_7041 to 7060	This study
ATCC25872-*aph*#4^{A1552} hybrid clones 1-20	ATCC25872 transformed with gDNA of strain A1552-*aph*#4 (#6914). 20 hybrid clones selected; intermediate $Strep^R$, Kan^R	MB_7061 to 7080	This study
ATCC25872-*aph*#5^{A1552} hybrid clones 1-20	ATCC25872 transformed with gDNA of strain A1552-*aph*#5 (#6915). 20 hybrid clones selected; intermediate $Strep^R$, Kan^R	MB_7081 to 7100	This study
ATCC25872-*aph*#6^{A1552} hybrid clones 1-20	ATCC25872 transformed with gDNA of strain A1552-*aph*#6 (#6916). 20 hybrid clones selected; intermediate $Strep^R$, Kan^R	MB_7101 to 7120	This study
ATCC25872-*aph*#7^{A1552} hybrid clones 1-20	ATCC25872 transformed with gDNA of strain A1552-*aph*#7 (#6917). 20 hybrid clones selected; intermediate $Strep^R$, Kan^R	MB_7121 to 7140	This study
ATCC25872-*aph*#8^{A1552} hybrid clones 1-20	ATCC25872 transformed with gDNA of strain A1552-*aph*#8 (#6918). 20 hybrid clones selected intermediate $Strep^R$, Kan^R	MB_7141 to 7160	This study
ATCC25872-*aph*#9^{A1552} hybrid clones 1-20	ATCC25872 transformed with gDNA of strain A1552-*aph*#9 (#6919). 20 hybrid clones selected; intermediate $Strep^R$, Kan^R	MB_7161 to 7180	This study
ATCC25872-*aph*#10^{A1552} hybrid clones 1-20	ATCC25872 transformed with gDNA of strain A1552-*aph*#10 (#6920). 20 hybrid clones selected; intermediate $Strep^R$, Kan^R	MB_7181 to 7200	This study
ATCC25872-*aph*#11^{A1552} hybrid clones 1-20	ATCC25872 transformed with gDNA of strain A1552-*aph*#11 (#6921). 20 hybrid clones selected; intermediate $Strep^R$, Kan^R	MB_7201 to 7220	This study
ATCC25872-*aph*#12^{A1552} hybrid clones 1-20	ATCC25872 transformed with gDNA of strain A1552-*aph*#12 (#6922). 20 hybrid clones selected; intermediate $Strep^R$, Kan^R	MB_7221 to 7240	This study
ATCC25872-*aph*#13^{A1552} hybrid clones 1-20	ATCC25872 transformed with gDNA of strain A1552-*aph*#13 (#6923). 20 hybrid clones selected; intermediate $Strep^R$, Kan^R	MB_7241 to 7260	This study
ATCC25872-*aph*#14^{A1552} hybrid clones 1-20	ATCC25872 transformed with gDNA of strain A1552-*aph*#14 (#6924). 20 hybrid clones selected; intermediate $Strep^R$, Kan^R	MB_7261 to 7280	This study
ATCC25872-*aph*#15^{A1552} hybrid clones 1-20	ATCC25872 transformed with gDNA of strain A1552-*aph*#15 (#6925). 20 hybrid clones selected; intermediate $Strep^R$, Kan^R	MB_7281 to 7300	This study
ATCC25872-*aph*#16^{A1552} hybrid clones 1-20	ATCC25872 transformed with gDNA of strain A1552-*aph*#16 (#6926). 20 hybrid clones selected; intermediate $Strep^R$, Kan^R	MB_7301 to 7320	This study
ATCC25872-*aph*#17^{A1552} hybrid clones 1-20	ATCC25872 transformed with gDNA of strain A1552-*aph*#17 (#6927). 20 hybrid clones selected; intermediate $Strep^R$, Kan^R	MB_7321 to 7340	This study
ATCC25872-*aph*#18^{A1552} hybrid clones 1-20	ATCC25872 transformed with gDNA of strain A1552-*aph*#18 (#6928). 20 hybrid clones selected; intermediate $Strep^R$, Kan^R	MB_7341 to 7360	This study

ATCC25872-*aph*#19^{A1552} hybrid clones 1-20	ATCC25872 transformed with gDNA of strain A1552-*aph*#19 (#6929). 20 hybrid clones selected; intermediate $Strep^R$, Kan^R	MB_7361 to 7380	This study
ATCC25872-*aph*#20^{A1552} hybrid clones 1-20	ATCC25872 transformed with gDNA of strain A1552-*aph*#20 (#6930). 20 hybrid clones selected; intermediate $Strep^R$, Kan^R	MB_7381 to 7400	This study
ATCC25872-*aph*#21^{A1552} hybrid clones 1-20	ATCC25872 transformed with gDNA of strain A1552-*aph*#21 (#6931). 20 hybrid clones selected; intermediate $Strep^R$, Kan^R	MB_7401 to 7420	This study
ATCC25872-*aph*#22^{A1552} hybrid clones 1-20	ATCC25872 transformed with gDNA of strain A1552-*aph*#22 (#6932). 20 hybrid clones selected; intermediate $Strep^R$, Kan^R	MB_7421 to 7440	This study
ATCC25872-*aph*#23^{A1552} hybrid clones 1-20	ATCC25872 transformed with gDNA of strain A1552-*aph*#23 (#6933). 20 hybrid clones selected; intermediate $Strep^R$, Kan^R	MB_7441 to 7460	This study
ATCC25872-*aph*#24^{A1552} hybrid clones 1-20	ATCC25872 transformed with gDNA of strain A1552-*aph*#24 (#6934). 20 hybrid clones selected; intermediate $Strep^R$, Kan^R	MB_7461 to 7480	This study
ATCC25872-*aph*#25^{A1552} hybrid clones 1-20	ATCC25872 transformed with gDNA of strain A1552-*aph*#25 (#6935). 20 hybrid clones selected; intermediate $Strep^R$, Kan^R	MB_7481 to 7500	This study
ATCC25872-*aph*#26^{A1552} hybrid clones 1-20	ATCC25872 transformed with gDNA of strain A1552-*aph*#26 (#6936). 20 hybrid clones selected; intermediate $Strep^R$, Kan^R	MB_7501 to 7520	This study
ATCC25872-*aph*#27(A1552) hybrid clones 1-20	ATCC25872 transformed with gDNA of strain A1552-*aph*#27 (#6937). 20 hybrid clones selected; intermediate $Strep^R$, Kan^R	MB_7521 to 7540	This study
ATCC25872-*aph*#28^{A1552} hybrid clones 1-20	ATCC25872 transformed with gDNA of strain A1552-*aph*#28 (#6938). 20 hybrid clones selected; intermediate $Strep^R$, Kan^R	MB_7541 to 7560	This study
ATCC25872-*aph*#29^{A1552} hybrid clones 1-20	ATCC25872 transformed with gDNA of strain A1552-*aph*#29 (#6939). 20 hybrid clones selected; intermediate $Strep^R$, Kan^R	MB_7561 to 7580	This study
ATCC25872-*aph*#30^{A1552} hybrid clones 1-20	ATCC25872 transformed with gDNA of strain A1552-*aph*#30 (#6940). 20 hybrid clones selected; intermediate $Strep^R$, Kan^R	MB_7581 to 7600	This study
ATCC25872-*aph*#31^{A1552} hybrid clones 1-20	ATCC25872 transformed with gDNA of strain A1552-*aph*#31 (#6941). 20 hybrid clones selected; intermediate $Strep^R$, Kan^R	MB_7601 to 7620	This study
ATCC25872-*aph*#32^{A1552} hybrid clones 1-20	ATCC25872 transformed with gDNA of strain A1552-*aph*#32 (#6942). 20 hybrid clones selected; intermediate $Strep^R$, Kan^R	MB_7621 to 7640	This study
ATCC25872-*aph*#33^{A1552} hybrid clones 1-20	ATCC25872 transformed with gDNA of strain A1552-*aph*#33 (#6943). 20 hybrid clones selected; intermediate $Strep^R$, Kan^R	MB_7641 to 7660	This study
ATCC25872-*aph*#34^{A1552} hybrid clones 1-20	ATCC25872 transformed with gDNA of strain A1552-*aph*#34 (#6944). 20 hybrid clones selected; intermediate $Strep^R$, Kan^R	MB_7661 to 7680	This study
ATCC25872-*aph*#35^{A1552} hybrid clones 1-20	ATCC25872 transformed with gDNA of strain A1552-*aph*#35 (#6945). 20 hybrid clones selected; intermediate $Strep^R$, Kan^R	MB_7681 to 7700	This study
ATCC25872-*aph*#36^{A1552} hybrid clones 1-20	ATCC25872 transformed with gDNA of strain A1552-*aph*#36 (#6946). 20 hybrid clones selected; intermediate $Strep^R$, Kan^R	MB_7701 to 7720	This study
ATCC25872-*aph*#37^{A1552} hybrid clones 1-20	ATCC25872 transformed with gDNA of strain A1552-*aph*#37 (#6947). 20 hybrid clones selected; intermediate $Strep^R$, Kan^R	MB_7721 to 7740	This study

ATCC25872-*aph*#38[A1552] hybrid clones 1-20	ATCC25872 transformed with gDNA of strain A1552-*aph*#38 (#6948). 20 hybrid clones selected; intermediate $Strep^R$, Kan^R	MB_7741 to 7760	This study
ATCC25872-*aph*#39[A1552] hybrid clones 1-20	ATCC25872 transformed with gDNA of strain A1552-*aph*#39 (#6949). 20 hybrid clones selected; intermediate $Strep^R$, Kan^R	MB_7761 to 7780	This study
ATCC25872-*aph*#40[A1552] hybrid clones 1-20	ATCC25872 transformed with gDNA of strain A1552-*aph*#40 (#6950). 20 hybrid clones selected; intermediate $Strep^R$, Kan^R	MB_7781 to 7800	This study
ATCC25872-*aph*#42[A1552] hybrid clones 1-20	ATCC25872 transformed with gDNA of strain A1552-*aph*#42 (#7922). 20 hybrid clones selected; intermediate $Strep^R$, Kan^R	MB_7951 to 7970	This study
ATCC25872-*aph*#32	ATCC25872 with *aph* cassette in position #32 on chr 2 (15kb upstream of T6SS large cluster); intermediate $Strep^R$, Kan^R	MB_6982	This study
A1552-*aph*#32[ATCC25872] hybrid clones 1-20	A1552 transformed with gDNA of strain ATCC25872-*aph*#32 (#6982). 20 hybrid clones selected; Rif^R, Kan^R	MB_9581 to MB_9600	This study
ATCC25872-*aph*#42	ATCC25872 with *aph* cassette in position #42 on chr 2 (upstream of *paar1/VCA0105*); intermediate $Strep^R$, Kan^R	MB_7995	This study
A1552-*aph*#42[ATCC25872] hybrid clones 1-20	A1552 transformed with gDNA of strain ATCC25872-*aph*#42 (#7995). 20 hybrid clones selected; Rif^R, Kan^R	MB_9601 to MB_9620	This study
A1552-T6SS [SNP45-T]	A1552 with SNP45 in intergenic region between *VCA0106* and *VCA0107* (*vipA*) converted from G to T by site-directed mutation; Rif^R	MB_9063	This study
A1552-T6SS [SNP45-C]	A1552 with SNP45 in intergenic region between *VCA0106* and *VCA0107* (*vipA*) converted from G to C by site-directed mutation; Rif^R	MB_9621	This study
A1552-T6SS [SNP45-A]	A1552 with SNP45 in intergenic region between *VCA0106* and *VCA0107* (*vipA*) converted from G to A by site-directed mutation; Rif^R	MB_9622	This study
ATCC25872-T6SS [SNP45-G]	ATCC25872 with SNP45 in intergenic region between *VCA0106* and *VCA0107* (*vipA*) converted from T to G by site-directed mutation; intermediate $Strep^R$	MB_9168	This study
A1552-*vipA-sfGFP*v2	A1552 carrying *vipA-sfGFP* translational fusion (version 2; without ATG at start of *sfGFP*); TransFLP method; Rif^R	MB_3909	[18]
A1552-T6SS [SNP45-T]-*vipA-sfGFPv2*	A1552-T6SS[SNP45-T] carrying *vipA-sfGFP* translational fusion (v2: without ATG at start of *sfGFP*), TransFLP method; Rif^R	MB_9521	This study
ATCC25872-*vipA-sfGFP*v2	ATCC25872 carrying *vipA-sfGFP* translational fusion (version 2; without ATG at start of *sfGFP*); TransFLP method; intermediate $Strep^R$	MB_9623	This study
ATCC25872- *vipA-sfGFP*v2 -T6SS [SNP45-G]	ATCC25872-*vipA-sfGFP*v2 with SNP45 in intergenic region between *VCA0106* and *VCA0107* (*vipA*) converted from T to G by site-directed mutation; intermediate $Strep^R$	MB_9624	This study
A1552Δ*tfoX*	A1552 with *tfoX* (*VC1153*) deleted; Rif^R	MB_45	[1]
A1552Δ*qstR*	A1552 with *qstR* (*VC0396*) deleted; Rif^R.	MB_600	[23]
A1552Δ*tfoY*	A1552 with *tfoY* (*VC1722*) deleted; Rif^R	MB_828	[18]
A1552Δ*vipB*	A1552 with *vipB* (*VCA0108*) deleted; Rif^R	MB_598	[4]
A1552Δ*vasK*	A1552 with *vasK* (*VCA0120*) deleted; Rif^R	MB_585	[4]
A1552Δ*hapR*	A1552 with *hapR* (*VC0583*) deleted; Rif^R	MB_3	[1]
A1552-T6SS [SNP45-T] Δ*tfoX*	A1552-T6SS[SNP45-T] with *tfoX* (*VC1153*) deleted; Rif^R	MB_9625	This study
A1552-T6SS [SNP45-T] Δ*qstR*	A1552-T6SS[SNP45-T] with *qstR* (*VC0396*) deleted; Rif^R	MB_9626	This study
A1552-T6SS [SNP45-T] Δ*tfoY*	A1552-T6SS[SNP45-T] with *tfoY* (*VC1722*) deleted; Rif^R	MB_9627	This study

A1552-T6SS [SNP45-T] Δ*vipB*	A1552-T6SS[SNP45-T] with *vipB* (*VCA0108*) deleted; Rif^R	MB_9628	This study
A1552-T6SS [SNP45-T] Δ*vasK*	A1552-T6SS[SNP45-T] with *vasK* (*VCA0120*) deleted; Rif^R	MB_9317	This study
A1552-T6SS [SNP45-T] Δ*hapR*	A1552-T6SS[SNP45-T] with *hapR* (*VC0583*) deleted; Rif^R	MB_9064	This study
A1552ΔIGR-ALL	A1552 with deletion of the intergenic region (396 bp) between *VCA0106* and *VCA0107* (*vipA*); 10 bp downstream of *VCA0106* and 25 bp upstream of *vipA* were kept; Rif^R	MB_9629	This study
A1552ΔIGR276	A1552 with deletion of 276 bp in intergenic region between *VCA0106* and *VCA0107* (*vipA*); Rif^R	MB_9630	This study
A1552ΔIGR336	A1552 with deletion of 336 bp in intergenic region between *VCA0106* and *VCA0107* (*vipA*); Rif^R	MB_9631	This study
A1552-T6SS [SNP45-T] ΔIGR276	A1552-T6SS[SNP45-T] with deletion of 276 bp in intergenic region between *VCA0106* and *VCA0107* (*vipA*); Rif^R	MB_9632	This study
A1552-T6SS [SNP45-T] ΔIGR336	A1552-T6SS[SNP45-T] with deletion of 336 bp in intergenic region between *VCA0106* and *VCA0107* (*vipA*); Rif^R	MB_9633	This study
A1552-T6SS [SNP45P-T] ΔIGR60	A1552-T6SS[SNP45-T] with deletion of 60 bp in intergenic region between *VCA0106* and *VCA0107* (*vipA*); Rif^R	MB_9634	This study
W10G	Environmental isolate (non-O1/non-O139) collected in 2004 in Waddell Creek (CA, USA)	MB_5537	[24]
SA3G	Environmental isolate (non-O1/non-O139) collected in 2004 in Old Salinas River (CA, USA)	MB_957	[24]
SA5Y	Environmental isolate (non-O1/non-O139) collected in 2004 in Old Salinas River (CA, USA)	MB_353	[24]
SL4G	Environmental isolate (non-O1/non-O139) collected in 2004 in San Lorenzo River (CA, USA); Amp^R	MB_955	[24]
SL4G-T6SS [SNP45-G]	SL4G with SNP45 in intergenic region between *VCA0106* and *VCA0107* (*vipA*) converted from T to G by site-directed mutation; Amp^R	MB_9635	This study
SL5Y	Environmental isolate (non-O1/non-O139) collected in 2004 in San Lorenzo River (CA, USA)	MB_954	[24]
SL5Y-T6SS [SNP45-G]	SL5Y with SNP45 in intergenic region between *VCA0106* and *VCA0107* (*vipA*) converted from T to G by site-directed mutation	MB_9636	This study
SO5Y	Environmental isolate (non-O1/non-O139) collected in 2004 in Soquel Creek (CA, USA)	MB_960	[24]
L6G	Wild-type; environmental isolate (non-O1/ non-O139) collected in 2004 in Lagunitas Creek (CA, USA); Amp^R.	MB_956	[24]
L6G-T6SS [SNP45-G]	L6G with SNP45 in intergenic region between *VCA0106* and *VCA0107* (*vipA*) converted from T to G by site-directed mutation; Amp^R.	MB_9637	This study
SL6Y	Wild-type; environmental isolate (non-O1/ non-O139) collected in 2004 in San Lorenzo River (CA, USA)	MB_953	[24]
SL6Y-T6SS [SNP45-G]	SL6Y with SNP45 in intergenic region between *VCA0106* and *VCA0107* (*vipA*) converted from T to G by site-directed mutation	MB_9638	This study
SP6G	Wild-type; environmental isolate (non-O1/ non-O139) collected in 2004 in San Pedro Creek (CA, USA).	MB_964	[24]
SP6G-T6SS [SNP45-G]	SP6G with SNP45 in intergenic region between *VCA0106* and *VCA0107* (*vipA*) converted from T to G by site-directed mutation	MB_9639	This study
SP7G	Wild-type; environmental isolate (non-O1/ non-O139) collected in 2004 in San Pedro Creek (CA, USA)	MB_952	[24]
W6G	Wild-type; environmental isolate (non-O1/ non-O139) collected in 2004 in Waddell Creek (CA, USA)	MB_354	[24]
W7G	Wild-type; environmental isolate (non-O1/ non-O139) collected in 2004 in Waddell Creek (CA, USA)	MB_962	[24]

E7G	Wild-type; environmental isolate (non-O1/ non-O139) collected in 2004 in Moss Landing Harbor (CA, USA)	MB_963	[24]
SA7G	Wild-type; environmental isolate (non-O1/ non-O139) collected in 2004 in Old Salinas River (CA, USA)	MB_959	[24]
SA10G	Wild-type; environmental isolate (non-O1/ non-O139) collected in 2004 in Old Salinas River (CA, USA)	MB_5539	[24]
C6706 (Strep^{S}) (original)	Wild-type; O1 El Tor Inaba collected in 1991 in Peru. Original isolate before introduction of streptomycin resistance mutation; non-mutated *luxO;* Strep^{S}	MB_4522	Gift from J. Mekalanos [19]
C6706-T6SS [SNP45-T]	C6706 with SNP45 in intergenic region between *VCA0106* and *VCA0107* (*vipA*) converted from G to T by site-directed mutation; Strep^{S}	MB_9640	This study
E7946	Wild-type; O1 El Tor Ogawa isolated in 197 in Bahrain. Strep^{R}	MB_2600	Lab stock; [25]
E7946-T6SS [SNP45-T]	E7946 with SNP45 in intergenic region between *VCA0106* and *VCA0107* (*vipA*) converted from G to T by site-directed mutation; Strep^{R}	MB_9641	This study
P27459	Wild-type; O1 El Tor Inaba isolated in 1976 in Bangladesh; Strep^{R}	MB_1504	[26]
P27459-T6SS [SNP45-T]	P27459 with SNP45 in intergenic region between *VCA0106* and *VCA0107* (*vipA*) converted from G to T by site-directed mutation; Strep^{R}	MB_9642	This study
DRC-193A	Wild-type; O1 isolated in 2011 in the Democratic Republic of Congo; Strep^{R}	MB_1954	[4]
DRC-193A -T6SS [SNP45-T]	DRC-193A with SNP45 in intergenic region between *VCA0106* and *VCA0107* (*vipA*) converted from G to T by site-directed mutation; Strep^{R}	MB_9643	This study
N16961-*hapR*^{Rep}	Wild-type; O1 El Tor Inaba isolated in 1975 in Bangladesh. *hapR* frameshift mutation repaired; Strep^{R}	MB_5663	[19]
N16961-*hapR*^{Rep}-T6SS [SNP45-T]	N16961-*hapR*^{Rep} with SNP45 in intergenic region between *VCA0106* and *VCA0107* (*vipA*) converted from G to T by site-directed mutation; Strep^{R}	MB_9644	This study
A1552-T6SS [SNP45-T]-chg[-10box]	A1552-T6SS[SNP45-T] with site-directed mutation (AA to GC) in -10 element in intergenic region between *VCA0106* and *VCA0107* (*vipA*); RifR, GentR	MB_9645	This study
A1552-Tn*tfoX*-strep (TnTfoX)	A1552 containing mini-Tn7-*araC*-P$_{BAD}$-*tfoX*-strep; RifR, GentR	MB_3420	[18]
A1552-chg[-10box]-Tn*tfoX*-strep (mut-10; TnTfoX)	A1552-Tn*tfoX*-strep with site-directed mutation (AA to GC) in -10 element in intergenic region between *VCA0106* and *VCA0107* (*vipA*); RifR, GentR	MB_9646	This study
A1552-Tn*qstR* (TnQstR)	A1552 containing mini-Tn7-*araC*-P$_{BAD}$-*qstR*; RifR, GentR	MB_5501	[27]
A1552-chg[-10box]-Tn*qstR* (mut-10; TnQstR)	A1552-Tn*qstR* with site-directed mutation (AA to GC) in -10 element in intergenic region between *VCA0106* and *VCA0107* (*vipA*); RifR, GentR	MB_9647	This study
A1552-Tn*tfoY*-strep (TnTfoY)	A1552 containing mini-Tn7-*araC*-P$_{BAD}$-*tfoY*-strep; RifR, GentR	MB_2978	[18]
A1552-chg[-10box]-Tn*tfoY*-strep (mut-10; TnTfoY)	A1552-Tn*tfoY*-strep with site-directed mutation (AA to GC) in -10 element in intergenic region between *VCA0106* and *VCA0107* (*vipA*); RifR, GentR	MB_9648	This study
E. coli			
TOP10	F- mcrA Δ(mrr-hsdRMS-mcrBC) φ80lacZΔM15 ΔlacX74 nupG recA1 araΔ139 Δ(ara-leu)7697 galE15 galK16 rpsL(StrR) endA1λ-.	MB_741	Invitrogen
TOP10-TnKan	TOP10 containing mini-Tn7-*aph* (Kan^{R}); Strep^{R}, Kan^{R}, Gent^{R}.	MB_4119	[18]

TOP10-TnGFP	TOP10 containing mini-Tn7-GFP; Strep^R, Cm^R, Gent^R.	MB_4482	This study
MC4100-TnGFP	MC4100 containing mini-Tn7-GFP; Strep^R, Cm^R, Gent^R.	MB_3930	This study
DH5α	F- endA1 glnV44 thi-1 recA1 relA1 gyrA96 deoR nupG φ80lacZΔM15 Δ(lacZYA-argF) U169 hsdR17 (rK$^-$ mK$^+$) phoA, λ-.	MB_736	[28]
S17-1λpir	Tpr Smr recA thi pro hsdR2M1 RP4:2-Tc:Mu:Kmr Tn7 (λpir); Strep^R.	MB_648	[29]
**Plasmids**			
pBR-FRT-Kan-FRT2	pBR322 derivative containing improved FRT-*aph*-FRT cassette, used as template for TransFLP; Amp^R, Kan^R.	MB_3782	[18]
pBR-FRT-Cat-FRT2	pBR322 derivative containing improved FRT-*cat*-FRT cassette, used as template for TransFLP; Amp^R, Cm^R.	MB_3783	[18]
pBR-flp	pBR322 derivative containing FLP+, λ cI857+, λ pR from pCP20 integrated into the *Eco*RV site of pBR322, used for FLP recombination; Amp^R	MB_1203	[2]
pGP704-Sac28	Suicide plasmid, *oriR6K sacB;* Amp^R	MB_694	[5]
pGP704-Sac28-[SNP45-T]	pGP704-Sac28 carrying a genome fragment resulting in a site-directed mutation in the SNP45 towards "T" located in the intergenic region between *VCA0106* and *VCA0107* (*vipA*); Amp^R	MB_9062	This study
pGP704-Sac28-[SNP45-G]	pGP704-Sac28 carrying a genome fragment resulting in a site-directed mutation in the SNP45 towards "G" located in the intergenic region between *VCA0106* and *VCA0107* (*vipA*); Amp^R	MB_9233	This study
pGP704-Sac28-[SNP45-C]	pGP704-Sac28 carrying a genome fragment resulting in a site-directed mutation in the SNP45 towards "C" located in the intergenic region between *VCA0106* and *VCA0107* (*vipA*); Amp^R	MB_9649	This study
pGP704-Sac28-[SNP45-A]	pGP704-Sac28 carrying a genome fragment resulting in a site-directed mutation in the SNP45 towards "A" located in the intergenic region between *VCA0106* and *VCA0107* (*vipA*); Amp^R	MB_9650	This study
pGP704-Sac28-SL6Y-[SNP45-G]	pGP704-Sac28 carrying a genome fragment resulting in a site-directed mutation in the SNP45 towards "G" located in the intergenic region between *VCA0106* and *VCA0107* (*vipA*) of strain SL6Y; Amp^R	MB_9651	This study
pGP704-Sac28-SP6G-[SNP45-G]	pGP704-Sac28 carrying a genome fragment resulting in a site-directed mutation in the SNP45 towards "G" located in the intergenic region between *VCA0106* and *VCA0107* (*vipA*) of strain SP6G; Amp^R	MB_9652	This study
p28-*tfoX* (pGP704-Sac28 -Δ*tfoX*)	pGP704-Sac28 carrying a gene fragment resulting in a deletion within *tfoX* (*VC1153*); Amp^R	MB_1013	[1]
pGP704-28-SacB-Δ*qstR*	pGP704-Sac28 carrying a gene fragment resulting in a deletion within *qstR* (*VC0396*). Amp^R.	MB_1118	[23]
pGP704-28-SacB-Δ*tfoY*	pGP704-Sac28 carrying a gene fragment resulting in a deletion within *tfoY* (*VC1722*). Amp^R.	MB_4116	[18]
pGP704-Sac28-Δ*vipB* (pGP704-28-SacB-ΔVCA0108)	pGP704-Sac28 carrying a gene fragment resulting in a deletion within *vipB* (*VCA0108*). Amp^R.	MB_1123	[4]
pGP704-Sac28-Δ*vasK* (pGP704-28-SacB-ΔVCA0120)	pGP704-Sac28 carrying a gene fragment resulting in a deletion within *vasK* (*VCA0120*). Amp^R.	MB_1124	[4]
p28-*hapR* (pGP704-Sac28 -Δ*hapR*)	pGP704-Sac28 carrying a gene fragment resulting in a deletion within *hapR* (*VC0583*). Amp^R.	MB_1038	[1]

pGP704-Sac28-ΔIGR276 [SNP45-G]	pGP704-Sac28 carrying a gene fragment resulting in a 276 bp deletion in the intergenic region between *VCA0106* and *VCA0107* (*vipA*) with a SNP45 as "G". 25 bp upstream of *vipA* were kept; Amp^R	MB_9653	This study
pGP704-Sac28-ΔIGR276 [SNP45-T]	pGP704-Sac28 carrying a gene fragment resulting in a 276 bp deletion in the intergenic region between *VCA0106* and *VCA0107* (*vipA*) with a SNP45 as "T". 25 bp upstream of *vipA* were kept; Amp^R.	MB_9654	This study
pGP704-Sac28-ΔIGR336 [SNP45-G]	pGP704-Sac28 carrying a gene fragment resulting in a 336 bp deletion in the intergenic region between *VCA0106* and *VCA0107* (*vipA*) with a SNP45 as "G". 25 bp upstream of *vipA* were kept; Amp^R	MB_9655	This study
pGP704-Sac28-ΔIGR336 [SNP45-T]	pGP704-Sac28 carrying a gene fragment resulting in a 336 bp deletion in the intergenic region between *VCA0106* and *VCA0107* (*vipA*) with a SNP45 as "T". 25 bp upstream of *vipA* were kept; Amp^R	MB_9656	This study
pGP704-Sac28-ΔIGR396 (Δfull)	pGP704-Sac28 carrying a gene fragment resulting in a 396 bp deletion of the intergenic region between *VCA0106* and *VCA0107* (*vipA*) – including SNP45. 10 bp downstream of *VCA0106* and 25 bp upstream of *vipA* were kept; Amp^R	MB_9657	This study
pGP704-Sac-Kan	Suicide plasmid, *oriR6K sacB*, Kan^R.	MB_6038	[6]
pGP704-Sac-Kan-L6G-[SNP45-G]	pGP704-Sac-Kan carrying a genome fragment resulting in a site-directed mutation in the SNP45 towards "G" located in the intergenic region between *VCA0106* and *VCA0107* (*vipA*) of strain L6G; Kan^R	MB_9658	This study
pGP704-Sac-Kan-SL5Y-[SNP45-G]	pGP704-Sac-Kan carrying a genome fragment resulting in a site-directed mutation in the SNP45 "G" located in the intergenic region between *VCA0106* and *VCA0107* (*vipA*) of strain SL5Y; Kan^R	MB_9659	This study
pGP704-Sac-Kan-SL4G-[SNP45-G]	pGP704-Sac-Kan carrying a genome fragment resulting in a site-directed mutation in the SNP45 towards "G" located in the intergenic region between *VCA0106* and *VCA0107* (*vipA*) of strain SL4G; Kan^R	MB_9660	This study
pGP704-Sac-Kan-ΔIG60bp [SNP45-T]	pGP704-Sac-Kan carrying a genome fragment resulting in a ~60bp deletion in the intergenic region between *VCA0106* and *VCA0107* (*vipA*) with a SNP45 as "T". 10bp downstream of *VCA0106* and 10bp upstream of *vipA* were kept; Kan^R	MB_9661	This study
pGP704-Sac-Kan-ΔIG60 [SNP45-G]	pGP704-Sac-Kan carrying a genome fragment resulting in a ~60bp deletion in the intergenic region between *VCA0106* and *VCA0107* (*vipA*) with a SNP45 as "G". 10bp downstream of *VCA0106* and 10bp upstream of *vipA* were kept; Kan^R	MB_9662	This study
pGP704-Sac-Kan-chg[-10box] [SNP45-T]	pGP704-Sac-Kan carrying a genome fragment resulting in a site-directed mutation in the -10 element (AA to GC) located in the intergenic region between *VCA0106* and *VCA0107* (*vipA*) with a SNP45 as "T"; Kan^R	MB_9663	This study
pGP704-Sac-Kan-chg[-10box] [SNP45-G]	pGP704-Sac-Kan carrying a genome fragment resulting in a site-directed mutation in the -10 element (AA to GC) located in the intergenic region between *VCA0106* and *VCA0107* (*vipA*) with a SNP45 as "G"; Kan^R	MB_9664	This study

*reference locus tags belong to reference strain N16961 according to [30].

Table S4. Statistics on PacBio whole-genome sequencing and genome assembly of strain ATCC25872.

	**ATCC25872**
**Strain ID**	MB#276
**BioSample ID**	SAMN13736322
**GenBank accession numbers (chr 1 / chr 2)**	CP047305 / CP047306
**Number of bases**	7.73 Gbp
**Number of reads**	414,427
**Mean read length**	18,897 bp
**Total number of contigs**	2 (chr1+chr2)
**Contig length after circularization**	2,945,491 bp (chr1)<br>1,072,958 bp (chr2)
**Total genome size**	4,018,449 bp
**Mean coverage**	1,796 x
**GC content**	47.8% (chr1)<br>46.9% (chr2)

Supplementary References

1. Meibom KL, Blokesch M, Dolganov NA, Wu C-Y, Schoolnik GK. (2005) Chitin induces natural competence in *Vibrio cholerae*. *Science* **310**:1824-7.
2. De Souza Silva O, Blokesch M. (2010) Genetic manipulation of *Vibrio cholerae* by combining natural transformation with FLP recombination. *Plasmid* **64**:186-95.
3. Blokesch M. (2012) TransFLP – a method to genetically modify *V. cholerae* based on natural transformation and FLP-recombination. *J Vis Exp* **68**:e3761.
4. Borgeaud S, Metzger LC, Scrignari T, Blokesch M. (2015) The type VI secretion system of *Vibrio cholerae* fosters horizontal gene transfer. *Science* **347**:63-7.
5. Meibom KL, Li XB, Nielsen AT, Wu CY, Roseman S, Schoolnik GK. (2004) The *Vibrio cholerae* chitin utilization program. *Proc Natl Acad Sci USA* **101**:2524-9.
6. Metzger LC, Matthey N, Stoudmann C, Collas EJ, Blokesch M. (2019) Ecological implications of gene regulation by TfoX and TfoY among diverse *Vibrio* species. *Environ Microbiol* **21**:2231-47.
7. Ben-Yaakov R, Salomon D. (2019) The regulatory network of *Vibrio parahaemolyticus* type VI secretion system 1. *Environ Microbiol* **21**:2248-60.
8. Keene ON. (1995) The log transformation is special. *Stat Med* **14**:811-9.
9. Schindelin J, Arganda-Carreras I, Frise E, Kaynig V, Longair M, Pietzsch T, et al. (2012) Fiji: an open-source platform for biological-image analysis. *Nat Methods* **9**:676-82.
10. Stutzmann S, Blokesch M. (2020) Comparison of chitin-induced natural transformation in pandemic *Vibrio cholerae* O1 El Tor strains. *Environ Microbiol* **22**:4149-66.
11. Sommer DD, Delcher AL, Salzberg SL, Pop M. (2007) Minimus: a fast, lightweight genome assembler. *BMC bioinformatics* **8**:64.
12. Seemann T. (2014) Prokka: rapid prokaryotic genome annotation. *Bioinformatics* **30**:2068-9.
13. Lo Scrudato M, Blokesch M. (2012) The regulatory network of natural competence and transformation of *Vibrio cholerae*. *PLoS Genet* **8**:e1002778.
14. Langmead B, Salzberg SL. (2012) Fast gapped-read alignment with Bowtie 2. *Nat Methods* **9**:357-9.
15. Anders S, Pyl PT, Huber W. (2014) HTSeq – A Python framework to work with high-throughput sequencing data. *bioRxiv preprint*:doi:10.1101/002824.
16. Love MI, Huber W, Anders S. (2014) Moderated estimation of fold change and dispersion for RNA-Seq data with DESeq2. *bioRxiv* **doi:10.1101/002832**.
17. Robinson JT, Thorvaldsdottir H, Winckler W, Guttman M, Lander ES, Getz G, et al. (2011) Integrative genomics viewer. *Nat Biotechnol* **29**:24-6.
18. Metzger LC, Stutzmann S, Scrignari T, Van der Henst C, Matthey N, Blokesch M. (2016) Independent Regulation of Type VI Secretion in *Vibrio cholerae* by TfoX and TfoY. *Cell Rep* **15**:951-8.
19. Van der Henst C, Vanhove AS, Drebes Dörr NC, Stutzmann S, Stoudmann C, Clerc S, et al. (2018) Molecular insights into *Vibrio cholerae*'s intra-amoebal host-pathogen interactions. *Nat Commun* **9**:3460.
20. Yildiz FH, Schoolnik GK. (1998) Role of *rpoS* in stress survival and virulence of *Vibrio cholerae*. *J Bacteriol* **180**:773-84.
21. Matthey N, Drebes Dörr NC, Blokesch M. (2018) Long-Read-Based Genome Sequences of Pandemic and Environmental *Vibrio cholerae* Strains. *Microbiol Resour Announc* **7**:e01574-18.
22. Aldova E, Laznickova K, Stepankova E, Lietava J. (1968) Isolation of nonagglutinable vibrios from an enteritis outbreak in Czechoslovakia. *J Infect Dis* **118**:25-31.

23. Lo Scrudato M, Blokesch M. (2013) A transcriptional regulator linking quorum sensing and chitin induction to render *Vibrio cholerae* naturally transformable. *Nucleic Acids Res* **41**:3644-58.
24. Keymer DP, Miller MC, Schoolnik GK, Boehm AB. (2007) Genomic and phenotypic diversity of coastal *Vibrio cholerae* strains is linked to environmental factors. *Appl Environ Microbiol* **73**:3705-14.
25. Miller VL, DiRita VJ, Mekalanos JJ. (1989) Identification of *toxS*, a regulatory gene whose product enhances ToxR-mediated activation of the cholera toxin promoter. *J Bacteriol* **171**:1288-93.
26. Pearson GD, Woods A, Chiang SL, Mekalanos JJ. (1993) CTX genetic element encodes a site-specific recombination system and an intestinal colonization factor. *Proc Natl Acad Sci USA* **90**:3750-4.
27. Jaskólska M, Stutzmann S, Stoudmann C, Blokesch M. (2018) QstR-dependent regulation of natural competence and type VI secretion in *Vibrio cholerae*. *Nucleic Acids Res* **46**:10619-34.
28. Yanisch-Perron C, Vieira J, Messing J. (1985) Improved M13 phage cloning vectors and host strains: nucleotide sequences of the M13mp18 and pUC19 vectors. *Gene* **33**:103-19.
29. Simon R, Priefer U, Pühler A. (1983) A broad host range mobilization system for *in vivo* genetic engineering: transposon mutagenesis in Gram negative bacteria. *Nat Biotechnol* **1**:784-91.
30. Heidelberg JF, Eisen JA, Nelson WC, Clayton RA, Gwinn ML, Dodson RJ, et al. (2000) DNA sequence of both chromosomes of the cholera pathogen *Vibrio cholerae*. *Nature* **406**:477-83.
